# Thickness of Intraretinal Layers in Patients with Type 2 Diabetes Mellitus Depending on a Concomitant Diabetic Neuropathy: Results of a Cross-Sectional Study Using Deviation Maps for OCT Data Analysis

**DOI:** 10.3390/biomedicines8070190

**Published:** 2020-07-02

**Authors:** Ruby Kala Prakasam, Aleksandra Matuszewska-Iwanicka, Dagmar-Christiane Fischer, Heidrun Schumann, Diethelm Tschöpe, Bernd Stratmann, Hans-Joachim Hettlich, Rudolf F. Guthoff, Oliver Stachs, Martin Röhlig

**Affiliations:** 1 Department of Ophthalmology, University Medical Center Rostock, Doberaner Str. 140, 18057 Rostock, Germany; ruby@lvpei.org (R.K.P.); rudolf.guthoff@med.uni-rostock.de (R.F.G.); oliver.stachs@med.uni-rostock.de (O.S.); 2 Professor Brien Holden Eye Research Centre, L V Prasad Eye Institute, Road 2 Banjara Hills, Hyderabad 500034, India; 3 Ruhr University Bochum, Johannes Wesling Hospital Minden, Hans-Nolte-Straße 1, 32429 Minden, Germany; aleksandra.matuszewska@gmail.com (A.M.-I.); hettlich@t-online.de (H.-J.H.); 4 Department of Pediatrics, University Medical Center Rostock, Ernst-Heydemann-Str. 8, 18057 Rostock, Germany; dagmar-christiane.fischer@med.uni-rostock.de; 5 Institute of Visual and Analytic Computing, University of Rostock, Albert-Einstein-Straße 22, 18059 Rostock, Germany; heidrun.schumann@uni-rostock.de; 6 Heart and Diabetes Center NRW, Ruhr University Bochum, Georgstraße 11, 32545 Bad Oeynhausen, Germany; dtschoepe@hdz-nrw.de (D.T.); bstratmann@hdz-nrw.de (B.S.)

**Keywords:** visual analytics, deviation maps, optical coherence tomography, intraretinal layers, type 2 diabetes mellitus

## Abstract

Optical coherence tomography (OCT) supports the detection of thickness changes in intraretinal layers at an early stage of diabetes mellitus. However, the analysis of OCT data in cross-sectional studies is complex and time-consuming. We introduce an enhanced deviation map-based analysis (MA) and demonstrate its effectiveness in detecting early changes in intraretinal layer thickness in adults with type 2 diabetes mellitus (T2DM) compared to common early treatment diabetic retinopathy study (ETDRS) grid-based analysis (GA). To this end, we obtained OCT scans of unilateral eyes from 33 T2DM patients without diabetic retinopathy and 40 healthy controls. The patients were categorized according to concomitant diabetic peripheral neuropathy (DN). The results of MA and GA demonstrated statistically significant differences in retinal thickness between patients and controls. Thinning was most pronounced in total retinal thickness and the thickness of the inner retinal layers in areas of the inner macular ring, selectively extending into areas of the outer macular ring and foveal center. Patients with clinically proven DN showed the strongest thinning of the inner retinal layers. MA showed additional areas of thinning whereas GA tended to underestimate thickness changes, especially in areas with localized thinning. We conclude that MA enables a precise analysis of retinal thickness data and contributes to the understanding of localized changes in intraretinal layers in adults with T2DM.

## 1. Introduction

Optical coherence tomography (OCT) [1] is an established method for detecting early signs of neurodegenerative changes in patients with diabetes mellitus (DM). Previous retinal OCT studies [2,3,4,5,6] have clearly demonstrated changes in the thickness profiles of distinct neuronal layers, primarily involving the retinal nerve fiber layer (RNFL), ganglion cell layer (GCL) and inner plexiform layer (IPL) in pediatric and adult DM patients at an early stage or even before any manifestation of clinical signs of microangiopathy (e.g., diabetic retinopathy (DR)). Such changes are thought to serve as biomarkers for the identification of patients at risk of developing DR. In this regard, software algorithms for the analysis of OCT data, in particular those for image segmentation [7], are a prerequisite for detailed analyses of individual retinal layers and thus are helpful for studying distinct ocular or systemic conditions. In general, the process of boundary identification and segmentation is followed by the generation of topographical color-coded thickness maps by measuring every single point of OCT data. While these color-coded thickness maps are spatially informative, it is hard to relate such data to the widely used early treatment diabetic retinopathy study (ETDRS) grid [8]. In fact, the established analysis approach of thickness profiles per retinal layer is currently based on averaged thickness values with respect to the ETRDS grid cells. The grid cells are defined by a central foveal ring with a 1 mm diameter, an inner macula ring (pericentral) with a 3 mm diameter and an outer macula ring (peripheral) with a 6 mm diameter. The inner and outer rings are divided into four quadrants (nasal, temporal, superior and inferior). To date, this grid-based analysis has been frequently used for the comparison of OCT findings between groups [3,9]. While this approach allows a quick overview of thickness changes in predefined macular regions, it consequently reduces the available amount of information. Hence, subtle and localized thickness differences within one cell are likely to be overlooked due to data averaging.

To address these issues, we developed an analysis software based on visual analytics (VA) methods [10,11]. VA is a special field in computer science that offers means for simplifying the analysis of multiple volumetric OCT datasets by combining automated algorithms and human abilities to visually detect trends and patterns in the data. Our VA software has been successfully tested on children with type 1 DM (T1DM) and age-matched controls [2]. Most importantly, we demonstrated a new map-based analysis using comparative color-coded deviation maps (DevMs), which helped us to understand the distribution of local thickness changes between two different study groups with greater spatial specificity. Statistical tests were directly applied to every single point on the DevMs to automatically highlight macular areas with significant aberration from the control.

In the present study, we extend our design with a novel method for precisely measuring irregular shaped areas of differences between study groups on DevMs (cf. Section 2.3) and discuss its advantages in detecting early neurodegenerative changes in the inner retina. We demonstrate our approach by comparing results from a conventional grid-based analysis (GA) to results from our enhanced map-based analysis (MA) of thickness changes in the inner retinal layers in patients with type 2 DM (T2DM) and controls of comparable age. Moreover, we show how our VA software can be used to flexibly define and compare subgroups of patients with diabetic peripheral neuropathy (DN).

## 2. Materials and Methods 

### 2.1. Inclusion and Exclusion Criteria for Patients and Controls

All T2DM patients were recruited from the Diabeteszentrum at Herz- und Diabeteszentrum Nordrhein-Westfalen, Germany, and the control group was assembled from colleagues at University Medical Center Rostock, Germany, on a voluntary basis. The study protocol was approved by the Institutional Review Board (Rostock University Medical Center Ethics Committee, Registration number: A 2015-0111 (dated 4 September 2015) with approved amendment (dated 26 February 2019)). All study procedures followed the tenets of the Declaration of Helsinki, and written informed consent was obtained from all subjects before enrolment.

Patients with a minimum age of 18 years, a clinical diagnosis of T2DM without DR, and an HbA1c ranging between 6.5% and 9.5% were eligible. Individuals suffering from T1DM, diabetic macular edema, proliferative DR, mental disabilities or a history of serious systemic illness (e.g., tumoral disease or neurological disorders except DN) were not eligible. Likewise, individuals with uveitis, optic nerve pathology, vitreous hemorrhage, contact lens wear (soft and rigid), symptomatic dry eyes, conjunctivitis at the time of examination, severe hyperopia, previous corneal or retinal surgery, media opacities such as dense cataracts, corneal opacities and epiretinal membranes were excluded.

Study examinations included the gathering of demographic and clinical data for the characterization of the disease (duration of the disease, mode of therapy, mean daily insulin dosages and mean HbA1c) by interview and/or medical chart review. All participants underwent comprehensive ophthalmic investigation, including unilateral retinal imaging (see below) and indirect ophthalmoscopy, to confirm the absence of DR. The neuropathy symptom score (NSS) and neuropathy deficient score (NDS) were used to test for DN. The patients with an NSS and/or NDS score ≤ 2 were classified as DN negative (DN_neg_) and the remaining patients as DN positive (DN_pos_).

### 2.2. Spectral Domain OCT

Randomly selected unilateral eyes of all participants underwent retinal imaging using spectra-domain OCT (SPECTRALIS HRA + OCT, Heidelberg Engineering GmbH, Heidelberg, Germany). The non-invasive procedures of OCT imaging were explained prior to examination and all participants were instructed to steadily fixate on an objective system of the device during the examination. The fixation was monitored by an active eye tracking system (TruTrack™) to ensure the precise registration of the OCT scans in the fundus image acquired with a confocal scanning laser ophthalmoscope (cSLO). Using the volume scan protocol, at least one volume scan was recorded for each participant. The scan presets were selected to closely match the area covered by the ETDRS grid. The volume scan size was defined to cover a rectangular area centered on the fovea and measuring at least 20° × 20° (5.9 × 5.9 mm). Per volume scan, at least 49 consecutive cross-sectional macular B-scans, each consisting of 16 averaged images (ART mean), with an interval minimum of 120 µm, were obtained.

All OCT images were acquired at a high-speed scanning modality with an axial and lateral resolution of 3.9 × 11 µm and with a signal quality of at least 27 dB. The device-specific software (Heidelberg Eye Explorer^®^, HEYEX version 6.5, Heidelberg Engineering GmbH, Heidelberg, Germany) was used for the retinal layer segmentation throughout the OCT datasets. Subsequently, the datasets were transferred to our VA software. The data transfer was established by means of a raw data export with the data format specification provided by the device manufacturer. Using the VA software, each dataset was screened to ensure data quality. In particular, data were checked for significant motion artifacts as well as for the accuracy and completeness of the automatically segmented retinal layers. The automated segmentations were verified by checking the generated thickness maps for artifacts (e.g., holes) and examining all individual B-scans for faulty layer boundary detection. Where possible, minor segmentation errors were corrected using the device software. Datasets which did not fulfill the quality criteria (e.g., very noisy or distorted B-scans and incomplete or failed retinal layer segmentation) were excluded. In this way, the best OCT scan per participant was selected. All selected scans were precisely aligned by determining the center of the fovea and the center of the optic disc as registration points. Finally, the center of the ETDRS grid was placed on the center of each fovea to allow accurate comparison during the interactive evaluation with the VA software.

### 2.3. Visual Analysis

The technical design of our VA software and a detailed description of its main features regarding the GA and MA of retinal OCT thickness data have been published previously [2,10,11]. Here, we utilized the VA software to evaluate the previously recorded OCT data of T2DM patients with and without DN relative to controls and to compare the results obtained with both methods.

**Figure 1:**
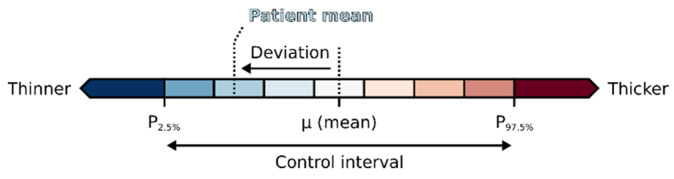
The color scale used to encode the deviation in mean thickness per grid cell or per map point. White indicates minimal patient deviation from the controls, and blue and red indicate negative (thinning) and positive (thickening) deviations, respectively. The degree of deviation is encoded by the color lightness, with the darkest colors representing values below or above the control interval.

OCT data were analyzed with ETDRS-based deviation grids [11] in GA and via DevMs [2] in MA. Basically, both methods demonstrate the absolute thickness difference of a retinal layer between groups, although with distinct differences regarding the presentation of the results. The colored grids offer a cell by cell overview on averaged thickness changes in predefined anatomical areas, whereas colored DevMs provide a point by point detail view on the actual shape and extent of thickness changes across different macular areas. The diverging color scale defined by the grids and maps explains the deviation in thickness of a retinal layer in comparison to reference data per grid cell or per map point (Figure 1). In the present study, the reference data were obtained from the thickness distribution of the control group, i.e., color reflects the thickness deviation with respect to the interval between the 2.5th and 97.5th percentiles of the control data. White indicates minimal deviation from the mean of the control group, and blue and red denote negative (thinning) or positive (thickening) deviations, respectively. The degree of deviation is encoded by the color lightness, with the darkest colors representing values below or above the 2.5th and 97.5th percentiles. The color-coded deviations were further tested for statistical significance (*p* < 0.05) at every grid cell and map point. We selected Student’s independent t-test for quantifying the comparison between T2DM patients and controls and Tukey’s honestly significant difference (HSD) test for multiple comparisons between the subgroups of DN_neg_ and DN_pos_ patients and controls. The resulting areas of significant differences were highlighted within the grids (orange cell borders and black cell labels) and within the maps (black outlines).

In addition, we used the VA software to automatically quantify the visualized differences in grids and maps. Based on the statistical test results, measurements were obtained of those grid cells in the GA and map areas in the MA that showed significant differences. Regarding the measurements in MA, a new method for the precise assessment of DevMs was integrated. The main idea was to use the ETDRS grid as a spatial frame of reference and to split the continuous significant areas on DevMs according to the anatomically distinct macular areas defined by its grid cells. Subsequently, the method involved measuring the significant fraction of the partitioned map areas within each superimposed ETDRS grid cell. This process included four automated steps: (i) point-based detection of statistically significant areas on DevMs, (ii) anatomy-oriented partitioning of the areas based on the ETDRS grid, (iii) measurement of only significant fractions of the areas within each grid cell and (iv) checking the fractions of areas against a coverage threshold. The coverage threshold was defined as the minimum percentage of grid cell area required to be covered by significant points on the DevMs. It was introduced to avoid the misinterpretation of particularly small areas in the maps. We selected a threshold of at least 10% coverage per ETDRS grid cell and the corresponding absolute mean deviation and standard error (MD ± SE) values in the MA were compared to the respective values of the GA.

## 3. Results

To test our VA software for OCT data analysis, we recruited a group of T2DM patients and assembled a control group of comparable age. The mean age of the T2DM patients (*n* = 33) and healthy controls (*n* = 40) was 60.4 ± 12.0 years and 56.0 ± 10.5 years, respectively. The anthropometric and clinical characteristics of the patients are summarized in Table 1. 

Using the VA software, GA and MA results for thickness deviation between patients and controls were generated. The concepts and algorithms involved in this process have been described previously [10,11]. The total thickness of the retina (TR) and the thickness of the RNFL, the GCL, the IPL and the inner nuclear layer (INL) were analyzed. Subsequently, patients were stratified according to the presence or absence of DN and the results of either subgroup were compared to the controls. For all comparisons, measurements only of statistically significant areas (*p* < 0.05) per ETDRS grid cell in GA and per fraction of those grid cells in MA were retrieved and reported as MD ± SE. Generally, both analysis methods revealed the presence of a considerable amount and extent of thinning primarily affecting the inner macular ring (IMR) and selectively extending into quadrants of the outer macular ring (OMR) in all investigated retinal layers, except for INL.

### 3.1. Comparison of T2DM Patients and Patient Subgroups to Controls for TR

**Figure 2:**
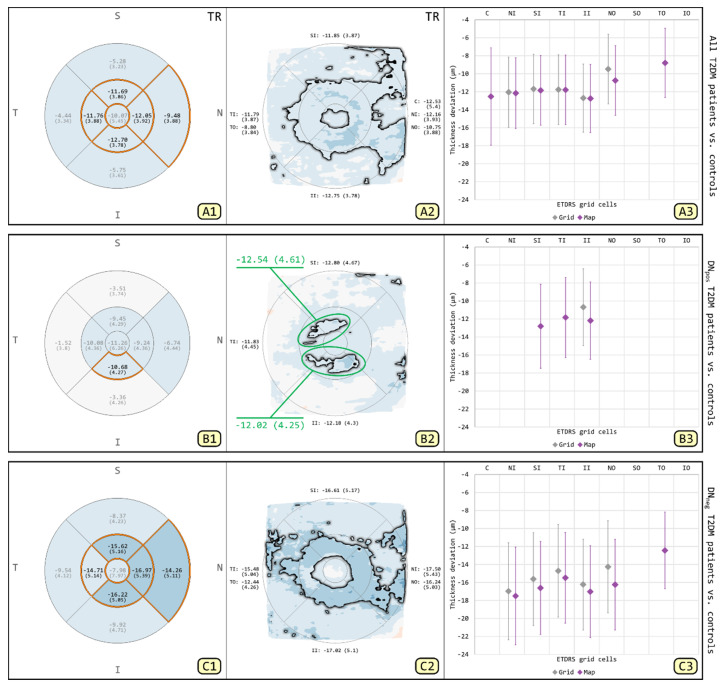
Comparisons for total thickness of the retina (TR) between all T2DM patients and controls (**A1**–**A3**), between subgroup of DN_pos_ T2DM patients and controls (**B1**–**B3**) and between subgroup of DN_neg_ T2DM patients and controls (**C1**–**C3**). The image columns correspond to results from grid-based analysis (GA) (**A1**,**B1**,**C1**), results from map-based analysis (MA) (**A2**,**B2**,**C2**) and their comparison using mean deviation and standard error (MD + SE) plots (**A3**,**B3**,**C3**). Color encodes thickness deviation (thinning: blue; thickening: red) and statistically significant differences (*p* < 0.05) are highlighted (grid cells: orange borders and black labels; maps: black outline). Two irregular shaped areas of interest were selected in one deviation map (DevM) and measured as individual defects (**B2**; green circles).

The results of GA and MA for TR are used to illustrate the differences between both methods (Figure 2). Comparison of the T2DM patients and controls for GA and MA revealed thinning of the TR primarily involving IMR, extending towards the nasal OMR. Within the subgroup of DN_pos_ T2DM patients, the thinning of the TR appears to be limited to the inferior IMR with GA, while MA detected an additional irregular shaped area of thinning involving the superior and temporal IMRs. By contrast, in DN_neg_ T2DM patients, GA and MA revealed thinning of the TR primarily involving the IMR, extending only towards the nasal OMR in GA but towards the nasal and temporal OMRs in MA. To highlight the differences between GA and MA, the respective results were compared using MD + SE plots (Figure 2(A3,B3,C3)). Overall, GA tended to underestimate thickness changes in ETDRS grid-defined areas, particularly in cells with localized thinning detected by MA. In addition, MA allowed the measuring of selected regions of interest as a single defect, such as the irregular shaped areas of localized thinning located at the inferior, temporal and superior IMRs on DevMs (Figure 2(B2), green circles).

### 3.2. Comparison of All T2DM Patients to Controls for RNFL, GCL and IPL

**Figure 3:**
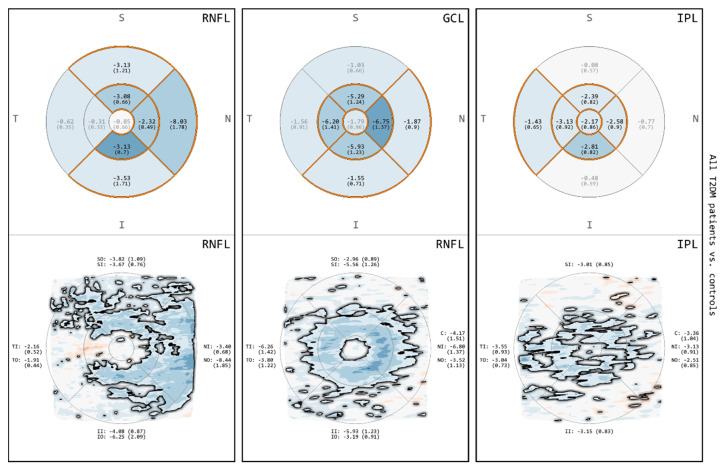
Comparisons for retinal nerve fiber layer (RNFL), ganglion cell layer (GCL) and inner plexiform layer (IPL) between all T2DM patients and controls. The image rows correspond to results from GA (**top**) and results from MA (**bottom**). Color encodes thickness deviation (thinning: blue; thickening: red) and statistically significant differences (*p* < 0.05) are highlighted (grid cells: orange borders and black labels; maps: black outline).

The results of the comparison between T2DM patients and controls with respect to the thickness of RNFL, GCL and IPL are depicted in Figure 3. For all of these layers, both GA and MA demonstrated areas of thinning primarily involving the IMR and selected quadrants of the OMR. In MA, the DevMs revealed a topographical overview of continuous or discrete thickness changes across different macular quadrants. The RNFL showed a greater amount and extent of thinning, extending towards the nasal OMR. The thinning in the GCL was more confined to the IMR, which exhibited a typical pattern of the normal foveal center (FC) surrounded by a circular band of thinning. The IPL exposed multiple discrete areas of thinning across the macular quadrants. Moreover, the FC of the IPL showed areas of thinning, which were not present in the RNFL or the GCL. 

### 3.3. Comparison between DN_pos_ T2DM Patients and Controls for RNFL, GCL and IPL

**Figure 4:**
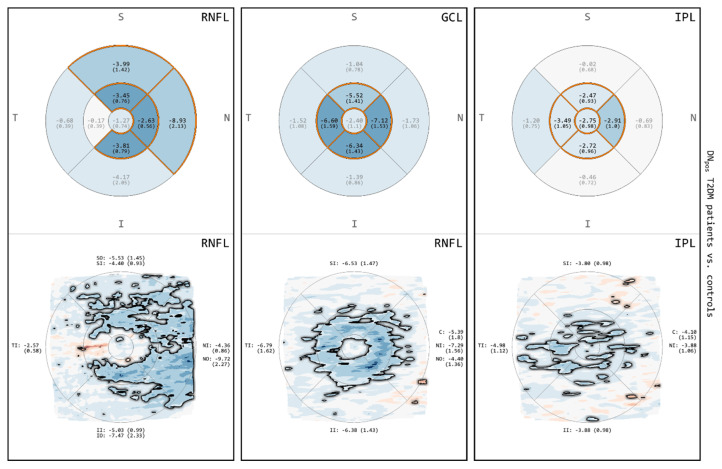
Comparisons for RNFL, GCL and IPL between subgroup of DN_pos_ T2DM patients and controls. The image rows correspond to results from GA (**top**) and results from MA (**bottom**). Color encodes thickness deviation (thinning: blue; thickening: red) and statistically significant differences (*p* < 0.05) are highlighted (grid cells: orange borders and black labels; maps: black outline).

The results of the comparison between DN_pos_ T2DM patients and controls for RNFL, GCL and IPL are shown in Figure 4. For all of these layers, the differences observed were similar to those obtained for the comparison between all T2DM patients and controls (Figure 3). Both GA and MA demonstrated areas of thinning involving the IMR and selected quadrants of the OMR. In MA, the RNFL showed a greater amount and extent of thinning extending towards the nasal OMR, the GCL exhibited a normal FC surrounded by a circular band of thinning, and the IPL exposed multiple discrete areas of thinning across the macular quadrants. The FC of the IPL again showed areas of significant thinning, which were not present in the RNFL or the GCL.

### 3.4. Comparison between DN_neg_ T2DM Patients and Controls for RNFL, GCL and IPL

**Figure 5:**
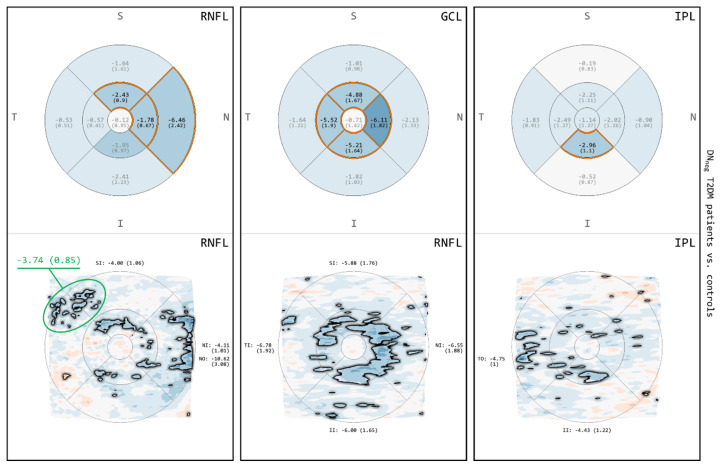
Comparisons for RNFL, GCL and IPL between subgroup of DN_neg_ T2DM patients and controls. The image rows correspond to results from GA (**top**) and results from MA (**bottom**). Color encodes thickness deviation (thinning: blue; thickening: red) and statistically significant differences (*p* < 0.05) are highlighted (grid cells: orange borders and black labels; maps: black outline). An interesting cluster of small areas of thinning spanning two grid cells was selected in one DevM and measured as a single defect (bottom left; green circle).

The results of the comparison between DN_neg_ T2DM patients and controls for RNFL, GCL and IPL are demonstrated in Figure 5. In contrast to the findings described above (Figure 3 and Figure 4), both GA and MA showed areas of thinning involving only parts of the IMR and OMR. In GA, the RNFL exhibited thinning involving the nasal and superior IMRs extending to the nasal OMR, the GCL showed a relatively greater amount and extent of thinning confined to the IMR, and the IPL exposed thinning of the inferior IMR. In MA, two areas of localized thinning in the RNFL (Figure 5; at 10 to 11 o’clock position in the OMR) and in the IPL (Figure 5; at temporal OMR) were noted in addition to those described with GA. In particular, the clustered multiple defects (small scattered areas of localized thinning) in the RNFL (Figure 5; bottom left; green circle) spanning both the superior and temporal OMRs were not identified in the GA (Figure 5; top left).

Furthermore, a few small and isolated areas of statistically significant thickening were observed only in MA in some DevMs of the RNFL, GCL and IPL, while in GA, only areas of thinning were demonstrated in all comparisons.

## 4. Discussion

In the present study, we demonstrated and compared results from both GA and MA to investigate early neurodegenerative changes in the retinas of patients with diabetes.

### 4.1. Comparison of Grid-Based and Map-Based Analyses

In general, the areas defined by the ETDRS grid cells are well suited to describing the thickness profile in four quadrants and three anatomically different rings (FC, IMR and OMR) of the central retina. Taking the natural shape of the retina into account, it is often acceptable to summarize and compare thickness measurements of quadrants within the IMR or OMR. However, considering the structural differences between the IMR and OMR, it is generally inappropriate to summarize and compare thickness measurements across macular rings. 

Previous retinal OCT studies [3,6,12,13,14] have adopted this grid-based analysis as a common way of presenting the GA results of group comparisons, either by deriving a mean thickness value for each individual grid cell or by averaging the mean thickness measures of four cells within one ring. This conventional approach grants an overview of thickness changes, but it bears the risk of losing information on subtle and localized changes within these generalized macular areas. The spatially precise DevMs have the ability to preserve such information. However, they have been solely applied to analyzing the thickness data of individuals and to measure single points on the maps in the past. Prior studies [4,12,15] include investigations of follow-up scans of an individual or relating the data of a single patient to predefined, often proprietary, normative data. Only recently, we have demonstrated a novel DevM design that, for the first time, supports point-based comparisons of layer thickness between different study groups and helps to analyze subtle and localized changes in the intraretinal layers in children with T1DM and matched controls [2]. Here, we extended our software with an additional feature that allows the accurate analysis of areas with statistically significant differences on DevMs, while also taking natural variations in retinal thickness in anatomically distinct macular areas into account. Compared to GA, which ends up with a mean thickness value for each ETDRS grid cell by averaging all normal and abnormal points, our enhanced MA provides more precise measurements of only abnormal points within each grid cell.

To avoid misinterpretation of the automated measurements, we introduced a coverage threshold as the minimum percentage of the grid cell area required to be covered by abnormal points on the DevMs (cf. Section 2.3). For example, it might be inappropriate to generalize a measurement of small and scattered areas of significant thinning (coverage threshold < 10%, *p* < 0.05) within a grid cell to the entire area of that cell (e.g., Figure 5, nasal and superior quadrants on DevM of IPL). At the same time, clusters of small areas of significant thinning, even with a coverage threshold of <10%, may appear relevant (e.g., Figure 5, superior-temporal OMR at around 10 o’clock on DevM of RNFL). We argue that these clusters need to be closely followed up in order to assess the thickness changes with regard to clinical findings and disease progression. For this purpose, our MA method enables the selection and measurement of any localized changes spanning two or more quadrants within a macular ring, such as the two irregular shaped areas of thinning on the DevM of TR (Figure 2(B2); green circles), measuring 12.54 ± 4.61 µm and −12.02 ± 4.25 µm, respectively. The measurement of such patterns might help to improve the understanding of a particular disease condition. As an example, in patients with diabetes or glaucoma, it might be interesting to compare the changes between the superior and inferior macular regions in order to investigate correlations related to the anatomical pattern of the RNFL bundle.

The MA method also enables direct comparisons between DevM measurements and corresponding grid measurements in GA. While both methods are similarly effective in representing general thickness changes in intraretinal layers, we expect that our new method will provide additional and probably meaningful details of the exact amount and extent of retinal changes. Furthermore, we evidently inferred a tendency of GA to produce false negative results due to the averaging of artifacts in the present study. For example, the irregular shaped area of significant thinning crossing the superior-temporal quadrants of the IMR was only detected in MA (Figure 2(B2)) but not as a significant cell in GA (Figure 2(B1)). Likewise, partial changes of the FC could be revealed in MA (Figure 2(A2)) but not in GA (Figure 2(A1)). Such foveal and macular involvements are a direct measure of central visual function. It is therefore essential to use an appropriate data analysis method for the accurate detection of changes in order to support early intervention.

### 4.2. Neurodegenerative Changes in Patients with T2DM

In general, the results from both GA and MA were comparable, and each method demonstrated significant thinning primarily involving the IMR in all except one of the investigated retinal layers, i.e., the INL. The thinning of the IMR also seemed to extend into the nasal OMR in TR and RNFL, which was particularly marked in RNFL. Our results are in line with those reported by others [3,4,13,16,17,18,19] in that the thickness of the inner retinal layers is reduced in patients with T2DM and this thinning is thought to be a marker of the beginning of neurodegenerative changes. While the majority of researchers evaluated the inner retinal layers, a number of studies [20,21] demonstrated morphological alterations in the choroidal and outer retinal layers in diabetes and DR. Therefore, it would be interesting to apply the VA software to study these parts of the retina in children and adults with diabetes. In the present study, we focused on the detection of spatial thickness changes relative to the presence or absence of DN. Our findings demonstrate the flexibility of our VA software regarding the utilization of interactive DevMs for comparison between controls and even subgroups of patients, i.e., those with and without DN. This approach revealed significant amounts of thinning in the RNFL and GCL, but there were no significant thickness changes in between subgroups (DN_pos_ T2DM patients compared to DN_neg_ T2DM patients) in any of the investigated retinal layers. However, the amount and extend of thinning detected in the DevMs of the RNFL and GCL was most prominent in the DN_pos_ T2DM patients. First of all, and in line with a recent study by Srinivasan, this indicates that DN is not restricted to the periphery but also affects the thickness of intraretinal layers [22,23]. Secondly, this strengthens the utilization of OCT as a measure for the sensitive monitoring of DM patients at risk of developing DN.

## 5. Conclusions

In conclusion, the enhanced deviation map-based analysis method is highly spatially specific and effective in detecting subtle and early neurodegenerative changes in the intraretinal layers. The importance and advantages of selecting an appropriate data analysis method to accurately measure retinal thickness changes is emphasized. As part of our continued research efforts, we plan to make the developed VA software available on a software sharing website in the future.

## Figures and Tables

**Table 1:** Clinical and demographic data of all type 2 diabetes mellitus (T2DM) patients and subgroups of T2DM patients with diabetic peripheral neuropathy (DN_pos_) or without (DN_neg_).

Characteristics	All T2DM Patients (*n* = 33)	DN_pos_ T2DM Patients (*n* = 21)	DN_neg_ T2DM Patients (*n* = 12)
**Age (yrs)**	60.4 ± 12.0	64.0 ± 9.0	54.0 ± 14.0
**Gender (male/female)**	21/12	15/6	6/6
**Height (cm)**	174 ± 10	176 ± 10	171 ± 9
**Weight (kg)**	99.3 ± 21.4	101.2 ± 21.4	95.9 ± 21.9
**BMI (kg/m^2^)**	32.7 ± 6.3	32.5 ± 5.2	33.1 ± 8.0
**Systolic blood pressure (mmHg)**	133 ± 16	134 ± 18	130 ± 10
**Diastolic blood pressure (mmHg)**	79 ± 11	79 ± 12	77 ± 6
**Duration of DM (yrs)**	13.3 ± 8.8	16.0± 9.6	8.7 ± 4.5
**Mean daily insulin dosage (IU/kg)**	0.515 ± 0.323 (*n* = 30)	0.516 ± 0.335 (*n* = 19)	0.512 ± 0.317 (*n* = 11)
**HbA1c (%)**	8.2 ± 0.8	8.2 ± 0.8	8.2 ± 0.9
**Neurologic disability score, NDS (pts)**	1.7 ± 1.9	2.7 ± 1.8	0
**Neurologic symptom score, NSS (pts)**	2.7 ± 1.9	3.9 ± 0.9	0.5 ± 0.9

## Abbreviations

OCT	Optical coherence tomography
VA	Visual Analytics
MA	Deviation map-based analysis
DevM	Deviation map
ETDRS	Early treatment diabetic retinopathy study
GA	ETDRS grid-based analysis
DM	Diabetes mellitus
T1DM	Type 1 diabetes mellitus
T2DM	Type 2 diabetes mellitus
DR	Diabetic retinopathy
DN	Diabetic peripheral neuropathy
DN_neg_	Patients without diabetic peripheral neuropathy
DN_pos_	Patients with diabetic peripheral neuropathy
FC	Foveal center
IMR	Inner macular ring
OMR	Outer macular ring
TR	Total thickness of the retina
RNFL	Retinal nerve fiver layer
GCL	Ganglion cell layer
IPL	Inner plexiform layer
INL	Inner nuclear layer
MD ± SE	Mean deviation and standard error
NSS	Neuropathy symptom score
NDS	Neuropathy deficient score
cSLO	Confocal scanning laser ophthalmoscope
HSD	Honestly significant difference
